# PIWIL2 induces c-Myc expression by interacting with NME2 and regulates c-Myc-mediated tumor cell proliferation

**DOI:** 10.18632/oncotarget.2327

**Published:** 2014-08-08

**Authors:** Youlin Yao, Chao Li, Xiaoyan Zhou, Yu Zhang, Yilu Lu, Jianhui Chen, Xulei Zheng, Dachang Tao, Yunqiang Liu, Yongxin Ma

**Affiliations:** ^1^ Department of Medical Genetics, State Key Laboratory of Biotherapy, West China Hospital, Sichuan University, Chengdu, China

**Keywords:** PIWIL2, c-Myc, NME2, Proliferation, F-actin

## Abstract

c-Myc serves as a crucial regulator in multiple cellular events. Cumulative evidences demonstrate that anomalous c-Myc overexpression correlates with proliferation, invasion and metastasis in various human tumors. However, the transcriptionally activating mechanisms responsible for c-Myc overexpression are complex and continue to be intangible. Here we showed that Piwi-Like RNA-Mediated Gene Silencing 2 (PIWIL2) can upregulate c-Myc via binding with NME/NM23 nucleoside diphosphate kinase 2 (NME2). PIWIL2 promotes c-Myc transcription by interacting with and facilitating NME2 to bind to G4-motif region within *c-Myc* promoter. Interestingly, in a c-Myc-mediated manner, PIWIL2 upregulates RhoA, which in turn induces filamentary F-actin. Deficiency of PIWIL2 results in obstacle for c-Myc expression, cell cycle progress and cell proliferation. Taken together, our present work demonstrates that PIWIL2 modulates tumor cell proliferation and F-actin filaments via promoting c-Myc expression.

## INTRODUCTION

c-Myc, encoded by proto-oncogene *Myc*, serves as a decisive regulator in various cytological functions such as proliferation, invasion and metastasis [1, 2]. Cumulate evidences explicitly depict that aberrant c-Myc overexpression is a common feature in various human tumors [3-7], however, the transcriptionally activating mechanisms responsible for c-Myc overexpression are complicated and remain elusive.

NME/NM23 nucleoside diphosphate kinase 2 (NME2), a ubiquitous enzyme isoform, transforms nucleoside diphosphates into triphosphates [8, 9], and well known for its homologue NME1 that is a tumor metastatic suppressor [10, 11]. Interestingly, a series of studies have shown that NME2 binds to a specific guanine-rich sequence known as G-quadruplex (G4-motif) located in *c-Myc* promoter. Thus, NME2 plays a role as a transcription factor activating c-Myc expression [12-14]. However, the underlying mechanisms controlling the transcriptional activation of *c-Myc* by NME2 continues to be unclear.

Piwi-Like RNA-Mediated Gene Silencing 2 (PIWIL2), alias HILI in human, belongs to Piwi protein subfamily, which functions in PIWI/piRNA pathway and plays crucial roles in gametogenesis [15, 16]. Tumor cells and germ cells share several characteristics such as exuberant proliferation [17]. Recent studies have demonstrated that PIWIL2 is expressed in various tumors [18-24].Our previous studies together with others’ also indicate that PIWIL2 contributes to proliferation and antiapoptosis in tumor cells [25, 26], however, the underlying mechanisms remain largely unclear.

Here we present an appealing association between PIWIL2 and c-Myc attributed to NME2. Our current study reveals that PIWIL2 raises c-Myc expression by binding to NME2, and subsequently enhances tumor cell proliferation and F-actin filaments.

## RESULTS

### PIWIL2 modulates the expression of c-Myc

**Figure 1:**
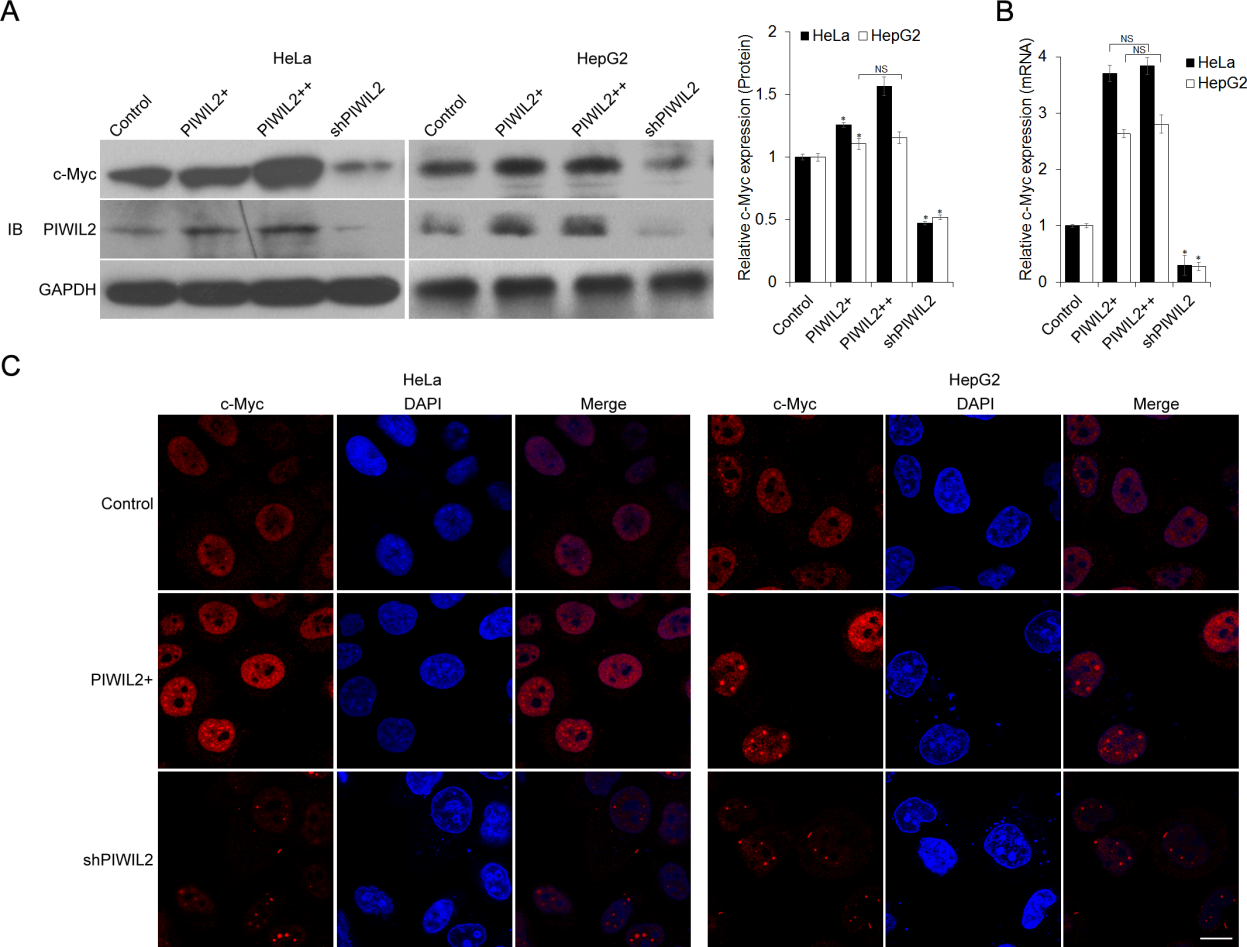
PIWIL2 alters c-Myc expression in tumor cells (A) PIWIL2 upregulates c-Myc expression at protein level. HeLa or HepG2 cells were transfected with indicated plasmids and harvested for Western blot analysis. (B) PIWIL2 upregulates c-Myc expression at mRNA level. RT-qPCR assay was performed and fold change was normalized by control to an arbitrary value of one. The results were presented as mean ± s.d. (n=3). *, P<0.05. NS, not significant. (C) Immunofluorescent staining of c-Myc in transfected cells with PIWIL2 expression constructs or shRNA expression vectors. HeLa and HepG2 cells were fixed, permeabilized and incubated with c-Myc primary antibodies and Cy3-labelled secondary antibodies (red) for c-Myc staining 48h after transfection. DAPI (blue), nucleus. Scale bar, 20μm.

To determine whether there is a correlation between PIWIL2 and c-Myc involving in the cellular processes, we first detected the possible change of c-Myc expression altered by PIWIL2. Expression constructs and shRNA expression vectors for PIWIL2 were transfected into HeLa or HepG2 cells. Western blot (WB) analysis revealed that c-Myc expression increased following the overexpression of PIWIL2 and, in contrast, c-Myc expression decreased in PIWL2-knockdowned cells (Figure 1A). Therefore, we asked whether the change of c-Myc expression at protein level was due to the change at mRNA level. A real-time quantitative PCR (RT-qPCR) analysis was performed and showed that c-Myc mRNA was upregulated by PIWIL2 overexpression, while knockdown of PIWIL2 markedly decreased the level of c-Myc mRNA both in HeLa and HepG2 cells (Figure 1B). As c-Myc is a transcription factor (TF) located in nucleus in tumor cells [1, 27], here we also detected the alteration of c-Myc caused by PIWIL2 expression by immunofluorescence (IF). Compared to control cells, c-Myc expression increased in PIWIL2-overexpressed cells and, conversely, weakened in PIWIL2-knockdowned cells (Figure 1C). Together, these results showed that PIWIL2 can upregulate c-Myc expression in tumor cells.

### PIWIL2 is involved in regulation of c-Myc by NME2

**Figure 2:**
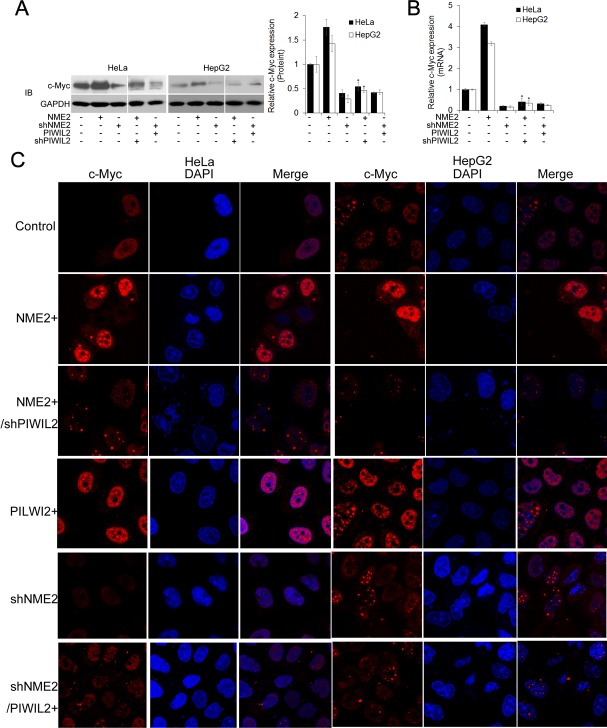
PIWIL2 is involved in regulation of c-Myc by NME2 (A) PIWIL2 knockdown suppresses NME2 induced c-Myc upregulation at protein level. HeLa or HepG2 cells were transfected with the indicated plasmids and harvested for Western blot analysis. (B) PIWIL2 knockdown suppresses NME2 induced c-Myc upregulation at mRNA level. Fold change was normalized by control to an arbitrary value of one. The results were presented as mean ± s.d. (n=3). *, P<0.05. NS, not significant. (C) Immunofluorescent staining of c-Myc in transfected cells. HeLa and HepG2cells were fixed, permeabilized and incubated with c-Myc primary antibodies and Cy3-labelled secondary antibodies (red) for c-Myc staining 48h after transfection. DAPI (blue), nucleus. Scale bar, 20μm.

NME2 has been identified as a transcriptional activator of c-Myc in existing researches [28, 29], and our result showed that NME2 upregulates c-Myc in both HeLa and HepG2 cells by using WB, RT-qPCR, and immunofluorescence analysis (Figure 2A-D). Therefore, we considered whether PIWIL2 is involved in NME2 inducing c-Myc transcription. Firstly, western blot assay was performed to determine whether PIWIL2 modulated the expression of NME2, which in turn elicited c-Myc transcription. The results showed that neither PIWIL2 nor NME2 affects the expression of the other (Supplementary FigureS1). Notably, further results showed that while PIWIL2 was knockdown, upregulation of c-Myc by NME2 at both mRNA and protein levels was suppressed (Figure 2A-D). These results suggested that PIWIL2 plays a role in NME2 transcriptionally activating c-Myc.

### PIWIL2 interacts with NME2

**Figure 3:**
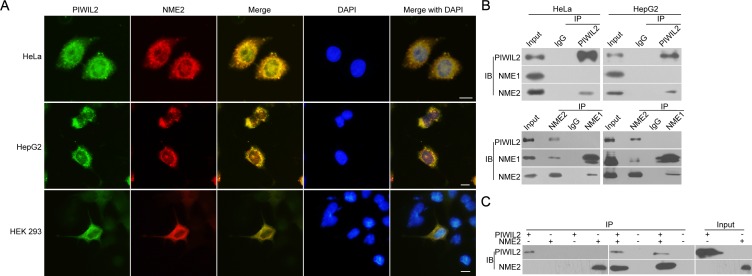
PIWIL2 interacts with NME2 (A) Co-localization of PIWIL2 and NME2 in HeLa or HepG2 cells, and in HEK293 cells co-transfected with expression vectors containing MYC-tagged PIWIL2 and HA-tagged NME2. Green (FITC), PIWIL2. Red (Cy3), NME2. Blue (DAPI), nucleus. Scale bar, 20μm. (B) Co-immunoprecipitation (coIP) assays showed that PIWIL2 interacts with NME2 but not NME1. (C) TNT® Quick Coupled Transcription/Translation System was employed to show the direct interaction of PIWIL2 and NME2.

Immunofluorescence assay showed that endogenous PIWIL2 and NME2 were expressed and overlapped in cytoplasm and nuclei in both HeLa and HepG2 cells (Figure 3A). Then, HEK293 cells were co-transfected by expression vectors of PIWIL2 and of NME2, and immunofluorescence assays showed that PIWIL2 overlaid with NME2 (Figure 3A). Further, co-immunoprecipitation assays were introduced to examine the interaction between PIWIL2 and NME2. As shown in Figure 3B, PIWIL2 interacts with NME2, but not NME1, a homologue of NME2, in HeLa and HepG2 cells. Similar results were also obtained by TNT®Quick Coupled Transcription/Translation Systems *in vitro* (Figure 3C). Together, these results indicate that PIWIL2 interacts with NME2.

### PIWIL2 facilitates NME2 binding to the c-Myc promoter

Some independent findings showed that NME2 binds to *c-Myc* promoter G-quadruplex (G4-motif) and induce c-Myc expression [14, 28, 29]. Based on our results above, we speculated whether the interaction between PIWIL2 and NME2 is involved in NME2 binding G4-motif and activating c-Myc expression.

**Figure 4:**
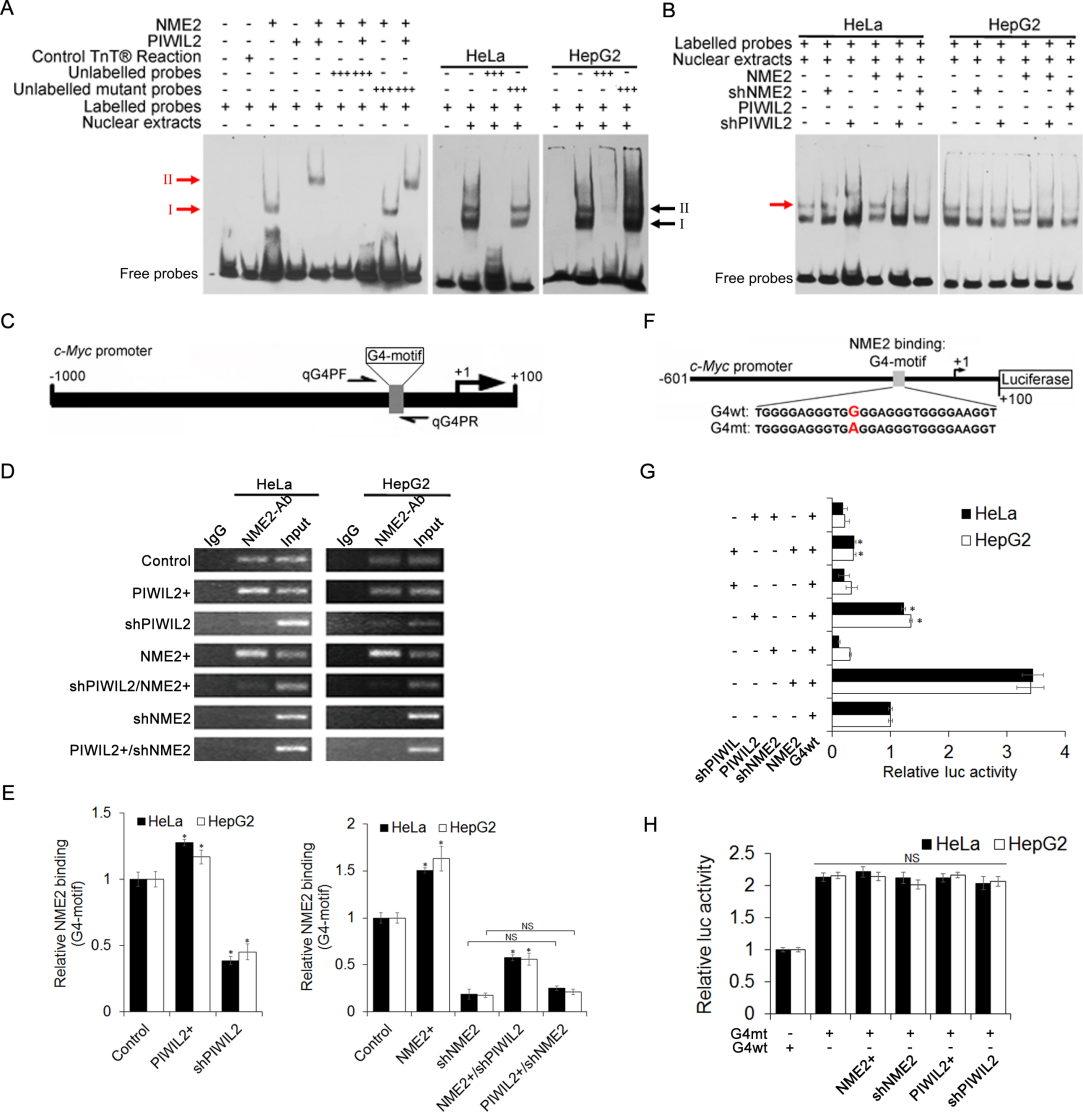
PIWIL2 facilitates NME2 binding toG4-motif region on c-Myc promoter (A) PIWIL2 shifts *c-Myc* G4-motif probe in presence of NME2.NME2 or PIWIL2 proteins was expressed by using TNT® Quick Coupled Transcription/Translation Systems. Control TNT® Reaction represented that pcDNA3.1 vector was used in TNT® assay *in vitro* as a negative control. Red arrows indicate protein/DNA complexes; black arrows indicate protein/DNA complexes in nuclear extracts. (B) EMSA assay showed that PIWIL2 knockdown suppresses the interaction between NME2 and *c-Myc* G4-motif probe in transfected cell nuclear extracts. Red arrow indicates specific protein/DNA complexes. (C) Details of G4-motif within *c-Myc* promoter and primers used in ChIP-qPCR. (D) ChIP assay was performed and analyzed by semi-qPCR. (E) ChIP assay was performed and analyzed by RT-qPCR. Results were normalized by control to an arbitrary value of one. (F) Details of c-Myc promoter showing wild type (G4wt) and mutant (G4mt) *c-Myc* G4-motif sequences for luciferase assays. (G) Luciferase assays were performed using *c-Myc* promoter (G4wt) in cells transfected with expression vectors or shRNA expression vectors for PIWIL2 or NME2 respectively. (H) Luciferase assays were performed using mutant (G4mt) *c-Myc*G4-motif promoter. (G) And (H) Relative promoter-luc activity was levelled by Renilla Luciferase activity, and data was normalized by control to an arbitrary value of one. The results were presented as mean ± s.d. (n=3). *, P<0.05. NS, not significant.

Using an electrophoretic mobility shift assay (EMSA), we observed that NME2 bound the biotin-labelled G4-motif DNA fragments, and yet PIWIL2 did not (Figure 4A, red arrow I). Results from binding reactions containing both NME2 and PIWIL2 expressed by TNT®Quick Coupled Transcription/Translation Systems showed that there was a further delayed band, indicating the NME2/PIWIL2-DNA complex (Figure 4A, red arrow II). The binding of protein complex to biotin-labelled G4-motif probe was competitively reduced by twenty-fold unlabeled G4-motif DNA (Figure 4A). However, when mutated sequence was used in excess amount, the binding was not reduced (Figure 4A). In addition, there were two retardant bands detected in both HeLa and HepG2 cell nuclear extracts, representing the protein bound to c-Myc promoter G4-motif (Figure 4A, black arrows I, II). Further, one of the retardant protein/DNA bands is undetected in NME2- or PIWIL2-knockdowned cells (Figure 4B, red arrow), indicating that this band represents the NME2/PIWIL2/G4-motif complex. Besides, when NME2 expression vector was co-transfected with shPIWIL2, the specific band indicating NME2/PIWIL2/G4-motif complex remained undetected (Figure 4B), suggesting that interaction between NME2 and G4-motif complex is PIWIL2-dependent in tumor cells.

To further confirm whether PIWIL2 contributes to NME2 binding to the G4-motif region of c-Myc promoter, we performed Chromatin immunoprecipitation (ChIP) assay with anti-NME2 antibodies. Following semi-qPCR and RT-qPCR analysis revealed that interaction between NME2 and G4-motif on *c-Myc* promoter was evidently enhanced in PIWIL2-overexpressed cells. Moreover, knockdown of PIWIL2 abates that interaction even when NME2 is overexpressed (Figure 4D, E). These results showed that PIWIL2 facilitates NME2 binding to *c-Myc* promoter.

We then investigated the ability of PIWIL2 to enhance NME2-mediated *c-Myc* transcription. Luciferase reporter vectors containing wild type or mutant *c-Myc* G4-motif were constructed and subjected to Dual-Luciferase reporter assay (Figure 4F). The relative Luciferase activity was significantly decreased with transfection of shPIWIL2 (Figure 4G). Earlier studies demonstrated that a single G to A substitution within G4-motif region offers about three-fold activity of *c-Myc* promoter in contrast to that of the wild type [14,30] (Figure 4F). Here we also showed that the relative luciferase activity of reporter vector containing mutant G4-motif (G4mt) was notably higher and unaltered by PIWIL2 knockdown (Figure 4H), suggesting that PIWIL2 enhances NME2 induced transcription activation of *c-Myc* via G4-motif.

Collectively, these findings suggest that PIWIL2 facilitates NME2-mediated transcription of c-Myc.

### c-Myc mediates PIWIL2-regulatedtumor cell proliferation and cell cycle progress

**Figure 5:**
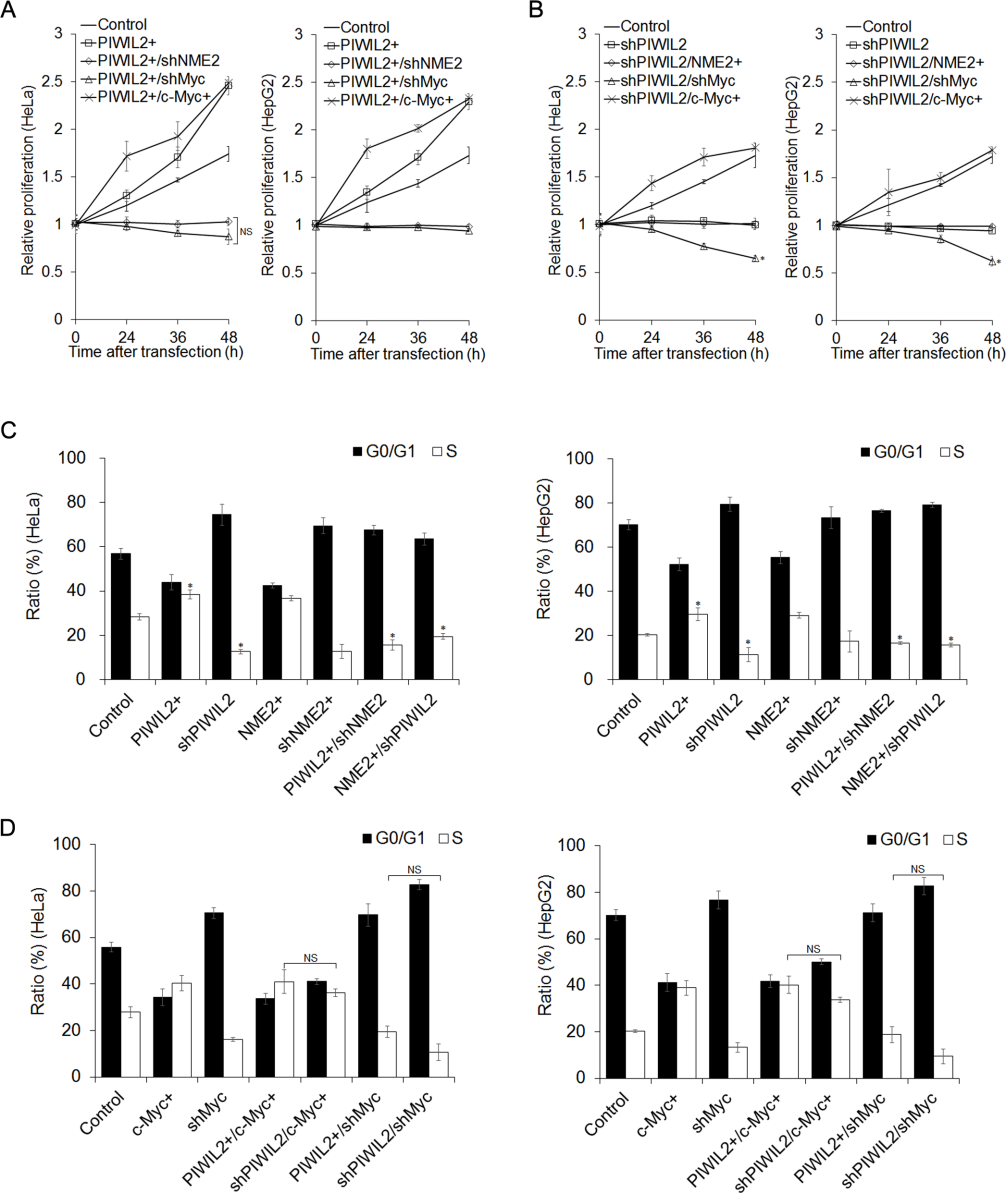
PIWIL2 can enhance cell proliferation and cell cycle progress via c-Myc (A) Cell Counting Kit-8 assay showed that c-Myc knockdown abates impact of PIWIL2 on cell proliferation. (B) c-Myc overexpression recovers impact of PIWIL2 knockdown on cell proliferation. (A) And (B) HeLa or HepG2 cells were transfected with various constructs for overexpression or knockdown by shRNA respectively. Cell proliferation was analyzed by Cell Counting Kit-8 assays in the indicated time. The relative proliferation was presented as fold change that was calculated by absorbance and normalized by control to an arbitrary value of one. (C) NME2 and PIWIL2 enhance cell cycle progress. (D) c-Myc is involved in PIWIL2 induced the entry of tumor cells from G0/G1 into S-phase. (C) And (D) HeLa or HepG2 cells were transfected with various constructs for overexpression or knockdown by shRNA respectively. After transfection, cells were stained with propidium iodide and the cell cycle was analyzed using flow cytometry. The results were presented as mean ± s.d. (n=3). *, P<0.05. NS, not significant.

Since PIWIL2 could promote tumor cells proliferation [25], we examined whether PIWIL2 regulates the proliferation trait of tumor cells through c-Myc. By performing cell counting kit-8 (CCK-8) assays, we observed that overexpression of PIWIL2 promotes cell proliferation both in HeLa and HepG2 cells (Figure 5A). Inversely, knockdown of PIWIL2 repressed proliferation of both cells (Figure 5B). Notably, knockdown of c-Myc by shRNA suppressed the proliferation induced by PIWIL2 (Figure 5A). Yet, c-Myc expression restored the proliferation impeded by PIWIL2 knockdown (Figure 5B). This result showed that PIWIL2 promotes tumor cell proliferation via c-Myc-mediated way.

Previous studies have demonstrated that c-Myc plays a crucial role in inducing cells entry from G0/G1 into S-phase [31, 32]. Due to PIWIL2 taking part in transcriptionally activating c-Myc observed above, we examined whether PIWIL2 promotes cell cycle progress in a c-Myc-mediated manner. Fluorescence-activated cell sorting (FACS) analysis showed that overexpression of PIWIL2 promotes the entry of cells from G0/G1 into S-phase, as compare to control cells (Figure 5C). Instead, PIWIL2-knockdown increased the number of G0/G1-phase cells markedly (Figure 5C). Interestingly, c-Myc knockdown baffled PIWIL2 induced cell cycle processing (Figure 5D). In addition, overexpression of c-Myc increased ratio of S-phase cells, which can be decreased by knockdown of PIWIL2 (Figure 5D). Taken together, these data suggested that c-Myc plays a part in PIWIL2-regulated tumor cell cycle progress and cell proliferation.

### PIWIL2 enhances F-actin filaments via c-Myc/RhoA pathway

**Figure 6:**
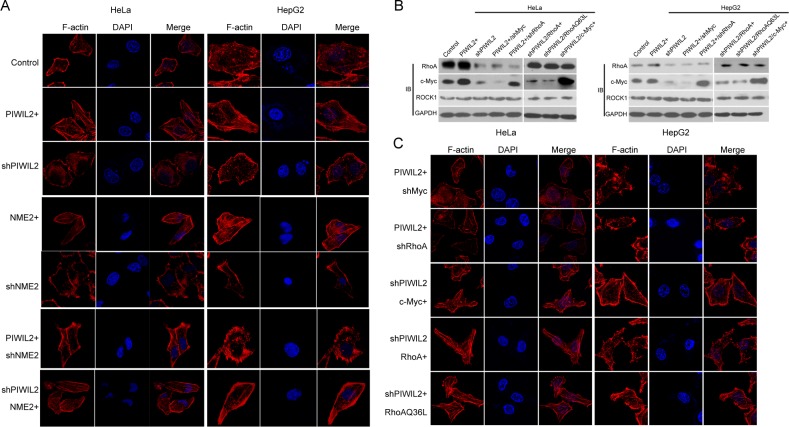
PIWIL2 induces F-actin filaments in tumor cells via c-Myc/RhoA pathway (A) PIWIL2 increases F-actin filaments in tumor cells. (B) PIWIL2 alters RhoA expression in a c-Myc-dependent manner. Transfected cells with indicated constructs were harvested for western blot analysis. (C) PIWIL2 induces F-actin stress fiber filaments via regulating c-Myc/RhoA pathway. (A) And (C) Transfected cells with indicated constructs were plated on coverslips for 24h prior to fixation, permeabilization, and staining for F-actin by TRITC-Phalloidine (Red), and then photoed via Olympus LSCM. Scale Bar, 20μm.

Previous studies have disclosed that c-Myc modulates filamentous F-actin via RhoA pathway [33-36]. We were interested in and surmised whether PIWIL2 affects F-actin through the pathway mentioned above. Due to c-Myc/RhoA pathway being conducive to tumor cell invasion and migration [33, 35], we first investigate whether PIWIL2 modulates the invasion and migration features of tumor cells by performing Transwell and wound healing assays. Results showed that PIWIL2-knockdowned cells displayed defective invasiveness (Supplementary Figure S2A). Besides, PIWIL2 overexpression resulted in enhanced migration in tumor cells (Supplementary Figure S2B). These observations reveal that PIWIL2 expression modifies tumor cell expansion in a certain way. Further, the F-actin filaments in HeLa and HepG2 cells were stained by TRITC-phalloidin, and observed under laser confocal microscopy (LSCM). Visibly, as shown in Figure 6A, PIWIL2 overexpression enhanced F-actin filaments, while F-actin filaments apparently weakened in PIWIL2-knockdowned cells. These results suggested that PIWIL2 affects F-actin filaments. RhoA is a critical regulator of filamentary F-actin formation [37, 38]. To gain insight into the possible relevance between PIWIL2 and RhoA, we next investigated whether PIWIL2 modulates the c-Myc/RhoA pathway that is critical for RhoA protein expression. Western blot analysis showed that PIWIL2 overexpression upregulated RhoA (Figure 6B). Otherwise, RhoA protein expression was reduced in PIWIL2-knockdowned cells, indicating that PIWIL2 regulates RhoA protein expression (Figure 6B). Notably, similar to the knockdown of RhoA, c-Myc knockdown diminished RhoA expression as well as filamentary F-actin in tumor cells induced by PIWIL2 (Figure 6B, C). Moreover, overexpression of c-Myc was able to restore RhoA protein expression and filamentary F-actin that were weakened by knockdown of PIWIL2 (Figure6B, C). Together, these findings revealed that PIWIL2 regulates F-actin filaments formation through c-Myc/RhoA pathway.

## DISCUSSION

The oncogene *c-Myc* is commonly activated in human tumors through various mechanisms. Its ungoverned expression contributes to tumor cell proliferation, invasion and metastasis [3, 7]. The mechanism for the upregulation of c-Myc in tumor cells is complicated and not completely clear. Previous studies have suggested that NME2 plays an indistinct role in transcriptional regulation. Unlike its homologue NME1 as a tumor suppressor, notably, it is regarded as an activator of c-Myc expression in human tumor cells [28, 29]. Recent studies have suggested that NME2 promotes *c-Myc* transcription by binding and disrupting the G-quadruplex (G4-motif) within *c-Myc* promoter [13, 14], which results in c-Myc expression inhibition [39]. However, the detailed mechanism how NME2 regulates c-Myc expression remains confused. These provoke our interests to explore the potential factors that interact with NME2 to activate c-Myc expression.

Previous studies demonstrate that PIWIL2 contributes to proliferation and antiapoptosis in tumor cells [25, 26]. Now by transfecting with expression constructs and shRNA we show that, analogous to NME2, also PIWIL2 raises c-Myc expression both in HeLa and HepG2 cells. (Figure 1). We then speculate whether there is a correlation between PIWIL2 and NME2 involved in triggering c-Myc expression. Western blot and RT-qPCR analysis as well as immunofluorescence assays indicated that PIWIL2 knockdown abates NME2 activated c-Myc expression (Figure 2), while PIWIL2 does not change the expression of NME2. Additional findings obtained here show that PIWIL2 is a new partner of NME2 in tumor cells, and the role of this interaction in c-Myc transcriptional regulation needs to be further elucidated. Our current study showed that PIWIL2 facilitates NME2 binding to the G4-motif sequence within *c-Myc* promoter, and subsequently induces *c-Myc* transcription. Previous studies have suggested that G4-motif region within the *c-Myc* promoter controls about 85% of the overall *c-Myc* transcription [29, 40, 41], and our present result showed that knockdown of PIWIL2 suppressed c-Myc significantly. Deficiency of either PIWIL2 or NME2 protein impairs the c-Myc expression, leading to defects in cell cycle progress and proliferation in tumor cells (Figure 5).

RhoA, a well-known small GTPase, regulates numerous biological behaviors such as F-actin filament formation or rearrangement, which is involved in tumor cell morphology and metastasis [33, 35, 37, 38]. Our present results revealed that PIWIL2 induces RhoA expression via regulating c-Myc, modulates F-actin filaments (Figure 6) and affects tumor cell invasion and migration (Figure S2).

**Figure 7:**
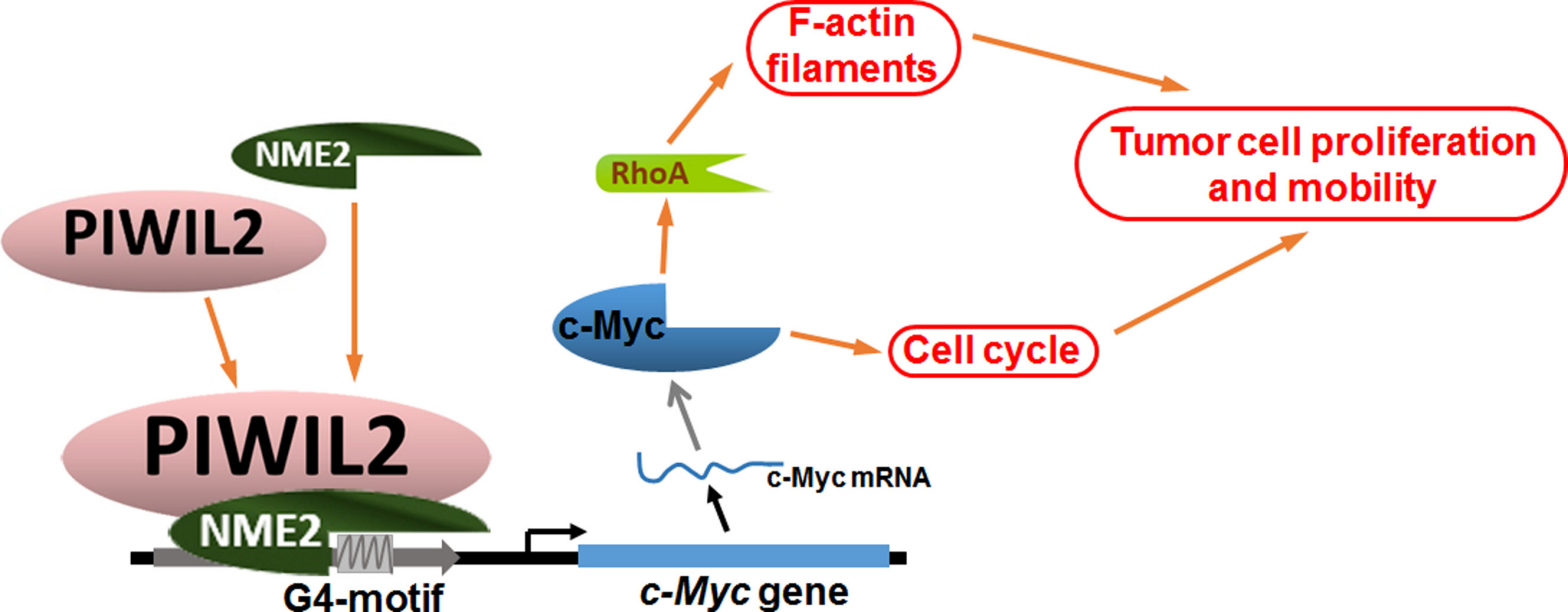
Model of PIWIL2 induction of c-Myc expression and modulation of c-Myc mediated cellular events

In summary, the current study reveals that PIWIL2 interacts with NME2 and facilitates NME2 binding to G4-motif to promote c-Myc expression, contributing to tumor cell proliferation and filamentary F-actin modulation via regulating RhoA expression (Figure 7). Our present work provides a novel insight into c-Myc overexpression occurring in tumor cells, and expands the knowledge of PIWIL2 in tumorigenesis.

## MATERIALS AND METHODS

### Cell culture and Transfection

Human cervical cancer cell line HeLa, hepatocellular carcinoma cell line HepG2 and HEK293 cell line were maintained in State Key Laboratory of Biotherapy and Cancer Centre of West China Hospital, Chengdu, PR China. The cells were cultured in Dulbecco's modified Eagle's medium (DMEM) with 10% fetal bovine serum (FBS) and penicillin–streptomycin (100 units/ml) at 37°C with 5% CO2. The transfection was performed in 6-well plates with jetPRIME™ (Cat. No. 114-15, Polyplus-transfection SA, France) following the manufacturers’ protocols. For expression analysis, a density of 1~ 2 x 10^5^ cells per well 24 h prior to transfection was seeded and harvested at 36h of culture for RT-qPCR analysis, or at 48h for Western blot analysis. For immunofluorescence staining, a low density of transfected cells (about 3 x 10^4^) was seeded and analyzed at indicated times. All following experiments were repeated three times unless stated otherwise.

### Constructs and shRNA

The expression constructs (pcDNA3.1+ vector) containing Myc-tagged PIWIL2 or 2×HA-tagged NME2, were generated and maintained by our laboratory. The cDNAs coding Flag-tagged c-Myc, Flag-tagged RhoA or Flag-tagged RhoAQ63L [42, 43] were synthesized and inserted into pcDNA3.1 (+) vector by GENEWIZ, Inc. PR China. To clone the certain c-Myc promoter, a 712 bp human c-Myc promoter fragment containing G4-motif region as described in previous studies [41,44] was amplified from the genomic DNA of HeLa cells and inserted into the pGL3 luciferase vector, marked as G4wt-Luc (reporter construct). The *c-Myc* mutant promoter (a single G to A substitution within G4-motif region) as described in earlier studies [14, 30] was generated by using the PCR-based method with the wild-type c-Myc promoter as a template. Then the amplified fragment was inserted into the pGL3 luciferase vector, marked as G4mt-Luc (reporter construct). Pairs of primers used in generating luciferase reporter constructs above were as follows: wild-type outer primers: mycP1-F: 5'- AGG CGG TAC CAA GTA TAC GTG GCA ATG CGT TGC -3', mycP1-R: 5'- AGG CAA GCT TCC GCC AAG CCT CTG AGA AGC -3'; mutated middle primers: G4m-UR: 5'- TGT GGA GGG TGG GGA AGG TGG GGA GGA GAC -3', G4m-DF: 5'- ACC TTC CCC ACC CTC CAC ACC CTC CCC ATA AGC GC -3'.

shRNA for PIWIL2, NME2, c-Myc, or RhoA was synthesized and cloned into shRNA expression vector pGPU6/GFP/Neo respectively by GenePharma Inc (Shanghai, PR China). The details of these shRNA were as follows: PIWIL2 shRNA: 5'- ACC GGC CUG GGU UGA ACU AAA -3' [26]; NME2 shRNA: 5'- AAU CCA GCA GAU UCA AAG CCA -3' [45]; c-Myc shRNA: 5'- GAU GAG GAA GAA AUC GAU G -3' [46]; RhoA shRNA: 5'- AUG GAA AGC AGG UAG AGU U -3' [47].

### Immunoblotting

Cells were harvested into lysis buffer (Bioteke, Beijing, PR China) containing protease inhibitor cocktail (Roche, Basel, Switzerland) after washing with PBS. Protein concentrations were determined by using BCA protein assay kit (Bioteke), and equal amounts of protein (15~30μg/sample) were subjected to sodium dodecyl sulfate–polyacrylamide gel electrophoresis (SDS-PAGE) and western blot analysis as described previously [26]. Antibodies used in the current study are described in Supplementary Table 1.

### Immunoprecipitation (IP)

Cells were lysed with the protein extraction reagent (Bioteke) supplemented with protease inhibitor cocktail (Roche). For PIWIL2-IP, cell lysates were incubated with 2μg PIWIL2 antibodies, or normal rabbit immunoglobulin G (IgG) as negative control. For NME2-IP, cell lysates were incubated with 2μg NME2 antibodies, or normal goat IgG as negative control. For NME1-IP, cell lysates were incubated with 2μg NME1 antibodies, or normal rabbit IgG as negative control. After an overnight incubated at 4°C, Protein A+G Agarose beads (40μl per reaction) were added (Cat. No. P2012, Beyotime, Jiangsu, PR China), and continually incubated at 4°C for 3h. Immunoprecipitated proteins were then subjected to SDS-PAGE and western blot analysis. Antibodies used in the IP are described in Supplementary Table 1.

### *In vitro* Binding Assays

In vitro protein binding assay was performed using TNT®Quick Coupled Transcription/Translation System (Cat. No. REFL1170, Promega, Madison, WI, USA) according to the manufacturer's instructions and previous studies [48]. In vitro expression assays for PIWIL2 and NME2 were carried out separately in 50μl volumes by adding 1μg of plasmid DNA. Followed these reactions, 2μl each of the PIWIL2 and NME2 reactions were used to analyze by Western blot with anti-PIWIL2 and anti-NME2. Subsequently, 30μl each of the PIWIL2 and NME2 reaction mixtures were used to co-IP assays elsewhere.

### Electric mobility shift assay (EMSA/Gel-shift)

EMSA was carried out using a Chemiluminescent EMSA Kit (Cat. No. GS009, Beyotime) as described previously [49]. Briefly, the following single-stranded 3'- biotinylated or unlabeled oligonucleotides were synthesized (BGI, PR China) and annealed to obtain DNA probes. G4-motif DNA (probe): 5'- TGG GGA GGG TGG GGA GGG TGG GGA AGG T -3', mutant G4-motif DNA (probe): 5'- TGA AGA GGG TGT GGA GGG TGA AGA AGG T -3'. PIWIL2 and NME2 proteins used in binding reactions were expressed by TNT®Quick Coupled Transcription/Translation System described above, and 2μl of each TNT reaction mixtures was used per binding reaction. For PIWIL2/NME2 binding assay, nuclear extracts were prepared using a Nuclear and Cytoplasmic Protein Extraction Kit (Cat. No. P0028, Beyotime) as described previously [50]. Binding reactions were performed in 10μl scale.

### Chromatin immunoprecipitation assay (ChIP) and Real-time quantitative PCR

ChIP assay was performed using a Chromatin Immunoprecipitation (ChIP) Assay Kit (Cat. No. P2078, Beyotime) as described previously [50]. Briefly, cells were fixed with 1% formalin for 10 min. Crosslinking was stopped by adding 125 mM glycine. Cells were washed, and then collected by brief centrifugation and lysed with SDS Lysis Buffer containing protease inhibitor cocktail (Roche). Sonication by using JY02-II Ultrasonic cell lyser (Ningbo, China) to fragment DNA to about 400 to 800 bp. After centrifugation, lysates were precleared with Protein A+G Agarose/Salmon Sperm DNA (Beyotmie). Precleared samples were diluted with the ChIP Dilution Buffer containing protease inhibitor cocktail. Then, NME2 and PIWIL2 were immunoprecipitated with anti-NME2 antibody (2μg) or anti-PIWIL2 antibody (2μg) and collected on Protein A+G Agarose. After serial washing with dialysis buffers, NME2-DNA complexes were eluted by Elution buffer (1% SDS, 0.1M NaHCO_3_). Eluted samples were mixed with 0.2 M NaCl and incubated at 65°C for 5h, then RNase A treated, and purified. PCR and RT-qPCR were performed using qG4PF primers: 5'- CTA CGG AGG AGC AGC AGA GAA AG -3', qG4PR primer: 5'- GTG GGG AGG GTG GGG AAG GT -3' as previously described [14]. ChIP samples were analyzed by RT-qPCR (CFX96 Touch, Bio-Rad Laboratories, Inc.) using SYBR®Premix Ex TaqTM II (Tli RNaseH Plus) (Code No. RR820A, TaKaRa)

### Luciferase reporter assay

Luciferase reporter assays were performed as previously described [26]. Briefly, HeLa or HepG2 cells were seeded into 12-well plates and transfected with the wild type (G4wt) or mutant (G4mt) *c-Myc* promoter luciferase reporter constructs using the jetPRIME™ (Polyplus). Co-transfections were performed with the respective expression constructs or shRNA expression vectors for PIWIL2 and NME2. Cells were harvested 24h post-transfection, and luciferase assay was performed with a Dual-Luciferase® Reporter Assay System kit (Cat.No. E1910) obtained from Promega using a Synergy 2 Multi-Mode Microplate Reader (BioTek). Relative promoter-luc activity was levelled by Renilla Luciferase activity.

### Immunofluorescence staining

Cells were fixed with 4% paraformaldehyde in PBS for 15min, permeabilized with 0.5% Triton X-100 in PBS for 15 min, and blocked with 1% BSA in PBS for 45 min at room temperature. Thereafter, cells incubated overnight at 4°C with primary antibodies and finally incubated with FITC- or Cy3-labelled secondary antibodies for 2h at RT. Each step was followed with 5 minutes washes in PBS three times. For F-action staining, followed the permeabilization, tetramethyl rhodamine isothiocyanate (TRITC)-conjugated phalloidin (Cat. No. P1951, Sigma-aldrich) was used to label cellular F-actin, and cell nuclei were counterstained with 4, 6-diamidino-2-phenylindole (DAPI) (Cat. No. D9542, Sigma). Cell samples were examined under a fluorescence inverse microscope (Olympus, Japan) or laser scanning confocal microscope (Olympus, Japan).

### Cell cycle assay

Cell cycles were examined by using a Cell Cycle Analysis Kit (Cat. No. C1052, Beyotime). Cells were fixed with 70% ethanol at 4°C overnight, and treated with RNaseA (0.02 mg/ml) in the dark at RT for 30 min, then stained with propidium iodide (PI) and analyzed by a COULTER EPICS XL flow cytometer (Beckman, USA) according to the manufacturer instruction.

### Cell counting kit-8 (CCK-8) assays

Cell proliferation was analyzed using a WST-8 Cell Counting Kit-8 (Cat. No. C0038, Beyotime) as previously described [51]. Cells were seeded in 96-well plates with 100μl of DMEM medium containing 10% FBS, and cultured in a humidified incubator (at 37°C, 5% CO_2_). Add10μl of the CCK-8 solution into each well of the plates in the indicated time after transfection. Incubate the plates for 2 hours in the incubator (at 37°C, 5% CO_2_). Measure the absorbance at 450 nm using a microplate reader. The relative proliferation was presented as fold change that was calculated by absorbance and normalized by control to an arbitrary value of one.

## SUPPLEMENTARY MATERIAL, FIGURES AND TABLE


