# Supplementary material for: PIWIL2 induces c-Myc expression by interacting with NME2 and regulates c-Myc-mediated tumor cell proliferation
Source: Oncotarget. 2014 Aug 8;5(18):8466–77. doi: 10.18632/oncotarget.2327 (PMC4226697; doi:10.18632/oncotarget.2327)
Supplement: Supplementary file 1 [file oncotarget-05-8466-s001.pdf]

PIWIL2 Induces c-Myc Expression by Interacting with NME2 and Regulates c-Myc-mediated Tumor Cell Proliferation

Supplementary Material

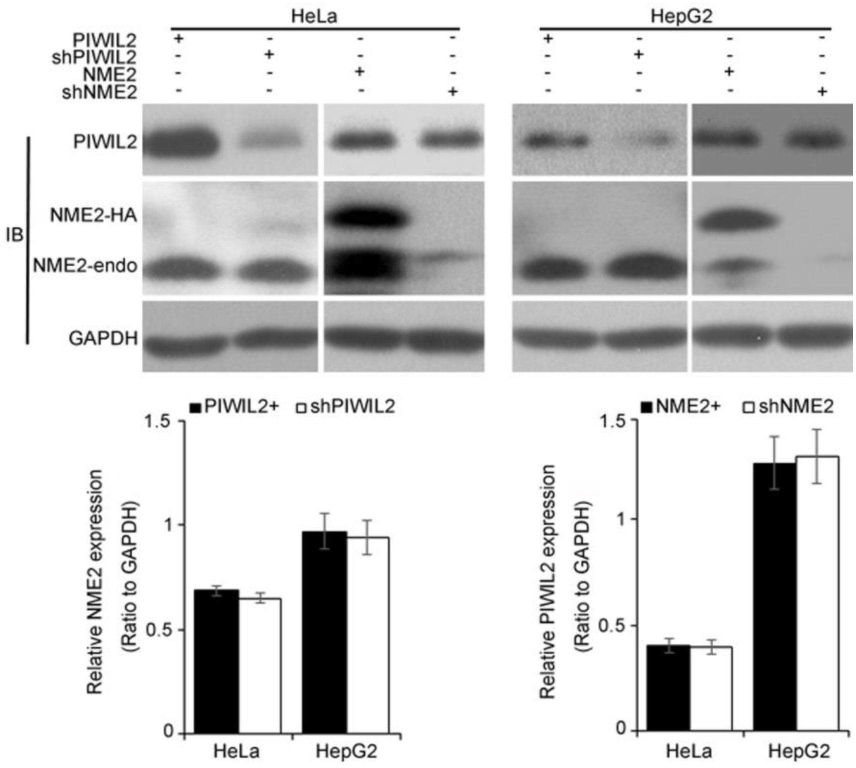

Supplementary Figure S1: Neither PIWIL2 nor NME2 can affect the expression of the other. No significant difference of PIWIL2 or NME2 was noted among samples of same cell type. Western blot analysis of PIWIL2 or NME2 expression. The relative changes in the protein level of PIWIL2 or NME2 was normalized by control to an arbitrary value of one. The results were presented as mean ± s.d. (n=3). NME2-2XHA, two HA epitope in series were fused at the C- end of NME2. NME2-endo, endogenous NME2 in indicated tumor cells.

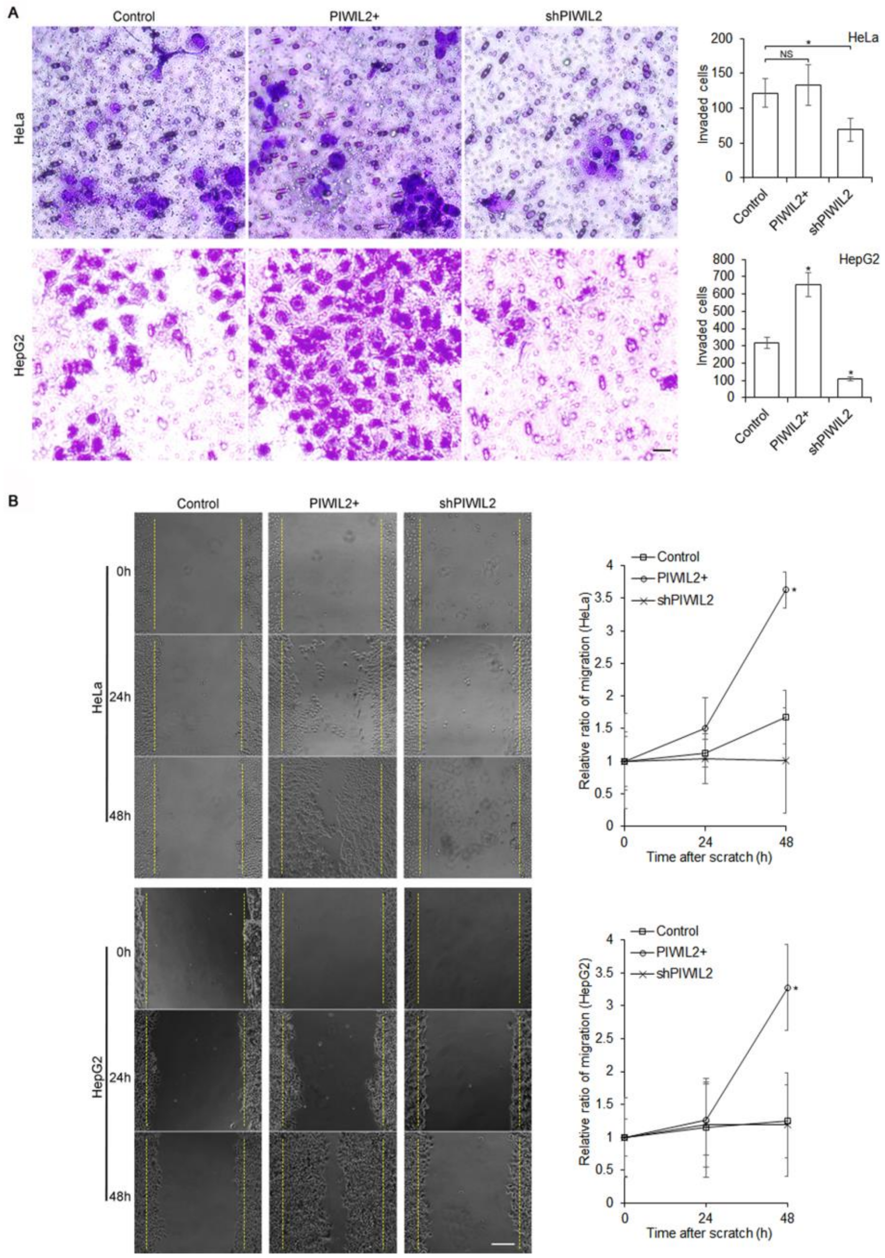

Supplementary Figure S2: PIWIL2 expression modulates the invasion and migration traits of tumor cells. (A) PIWIL2 knockdown weakens tumor cell invasion. Using a Cell Invasion Assay Kit with 8μm pore size polycarbonate inserts (Cat. No. ECM550, Millipore, Germany) as previously described [59], cell Invasion (Transwell) assay was performed to test PIWIL2 contributing to the invasive trait of tumor cells. Briefly, Add 500 µl of media containing 10% FBS to the lower chamber. Add 300 µl of prepared cells (1×10^5cells) suspended in serum free media into to each

insert. After48 h culture, cells on the upper side of the membrane were then removed, whereas the cells that migrated through the membrane and clung to the underside were fixed, and stained with the staining solution for 20 minutes at RT. Cell numbers were counted in five separate fields by microscopy. The quantified results were presented as the mean ± s.d. Scale bar, 40μm. **(B)** A role of PIWIL2 expression in migration of tumor cells were examined by wound-scratch (healing) assay. Transfected and control cells were scraped with a 200μl pipette tip. After being washed with PBS and serum-free DMEM medium, cells were incubated at 37℃in DMEM medium containing 2% FBS. The migration of cells was observed at the indicated times with an inverted microscope and photographed. The relative migration rate was quantified with measurements of the gap size during the culture, and normalized by control to an arbitrary value of one. The results were presented as mean ± s.d. (n=3). *, $P<0.05$. NS, not significant. Scale bar, 100μm.
